# Supplementary material for: Modified Hyaluronic Acid-Laminin-Hydrogel as Luminal Filler for Clinically Approved Hollow Nerve Guides in a Rat Critical Defect Size Model
Source: Int J Mol Sci. 2021 Jun 18;22(12):6554. doi: 10.3390/ijms22126554 (PMC8235360; doi:10.3390/ijms22126554)
Supplement: Supplementary file 1 [file ijms-22-06554-s001.zip › ijms-1249499-supplementary.pdf]

Supplementary figures

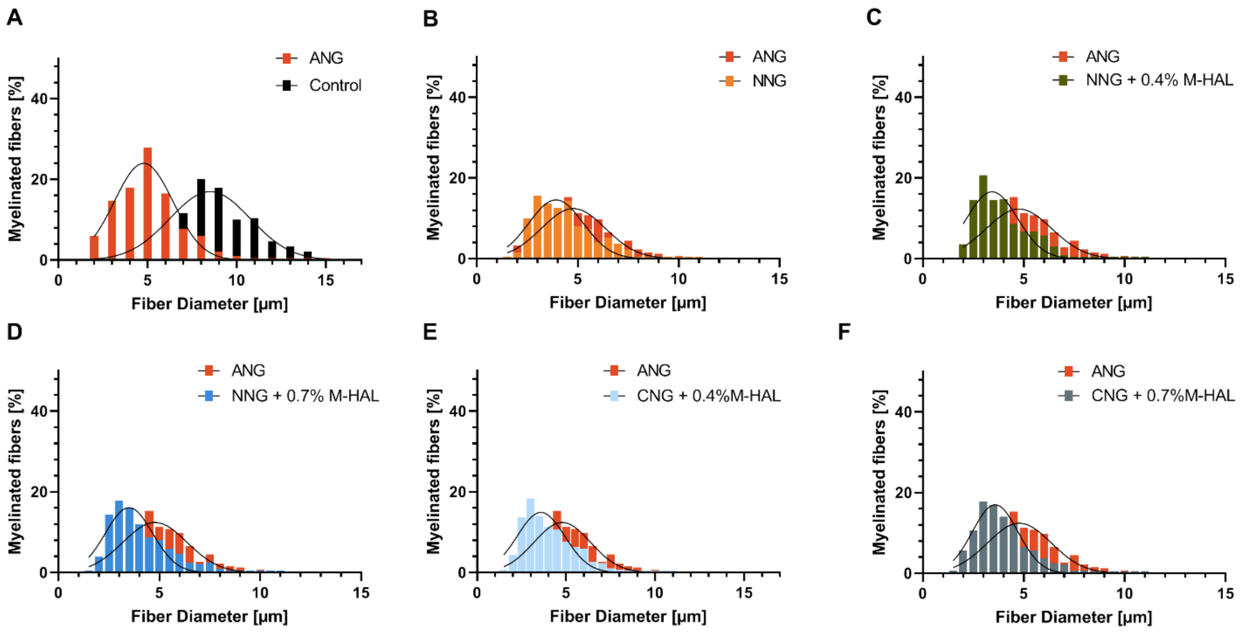

Figure S1. Graphical presentation of results from the current study of the morphometrical analysis of regenerated myelinated axons distal to the grafts. Bar graphs show the percentile distribution of nerve fiber diameters and the curves (black) were fitted to data using nonlinear regression (Gaussian equatation).. Values were derived from the morphometrical analysis of semi-thin cross-sections through distal nerve segments harvested at 120 days after reconstruction surgery. (A) Depicts the left shift in fiber diameter occurring after nerve reconstruction with the gold standard autograft (ANG, $n = 5$) in comparison to non-lesioned control nerve segments (Control, $n = 3$). (B-F) Depict a further left shift in fiber diameter for the comparison of myelinated fibers regenerated through ANG with all other graft types investigated in the current study. (B) Empty collagen-based nerve guide, NNG: $n = 4$; (C) NNG+0.4%M-HAL (modified hyaluronic acid-laminin gel), $n = 6$; (D) NNG+0.7%M-HAL: $n = 6$; € chitosan-based nerve guide (CNG) 0.4%M-HAL: $n = 4$; (F) CNG+0. 7%M-HAL: $n = 4$. Interestingly, myelinated axons regenerated through empty NNG or gold-standard ANG grafts displayed almost similar percentile nerve fiber diameter distributions.

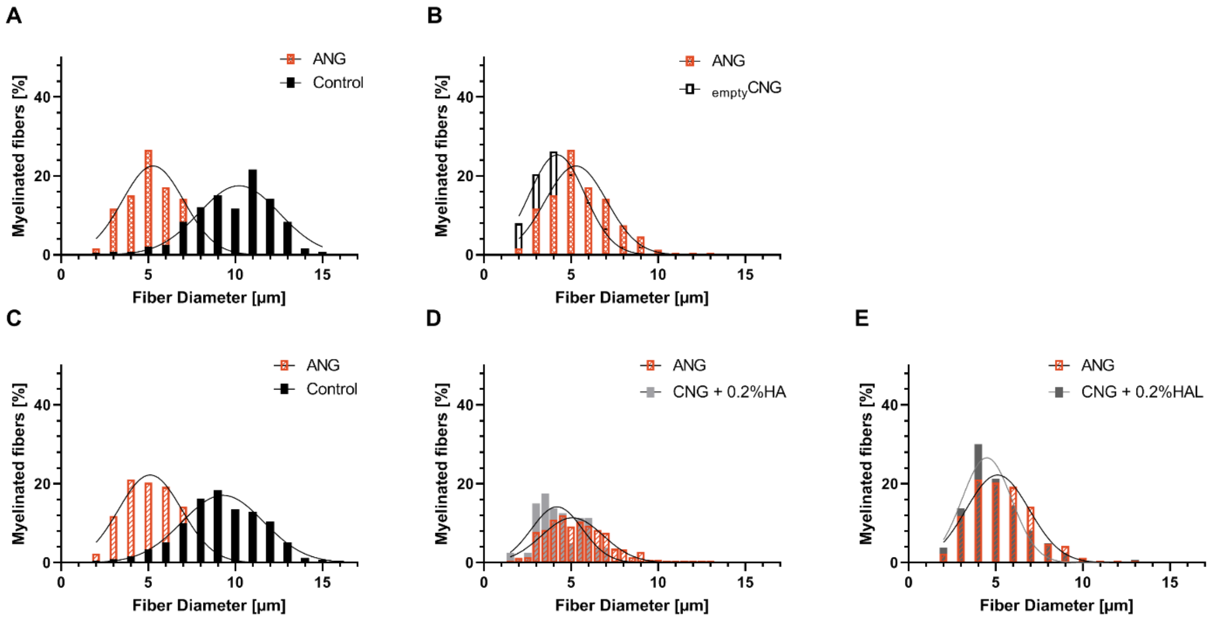

Figure S2. Graphical presentation of the results from re-evaluating our own previous data in morphometrical analysis of regenerated myelinated axons distal to the grafts. Bar graphs show the percentile distribution of nerve fiber diameters and the curves (black) were fitted to data using nonlinear regression (Gaussian equatation).. Values were derived from re-analysis of data published in *Stößel et al., 2018* (A) and re-analysis of data published in *Dietzmeyer et al., 2020* (B). The gold standard ANG was compared to the control and experimental groups. The number of evaluated animals was (A) control: n = 3; ANG: n = 3; CNG: n = 3, and (B) control: n = 6; ANG: n = 6; CNG+0.2%HA: n = 1; CNG+0.2%HAL: n = 2.
